# Evaluation on Radiometric Capability of Chinese Optical Satellite Sensors

**DOI:** 10.3390/s17010204

**Published:** 2017-01-22

**Authors:** Aixia Yang, Bo Zhong, Shanlong Wu, Qinhuo Liu

**Affiliations:** 1 State Key Laboratory of Remote Sensing Science, Institute of Remote Sensing and Digital Earth, Chinese Academy of Sciences, Beijing 100101, China; yangax@radi.ac.cn (A.Y.); wsl0579@163.com (S.W.); 2 College of Resource and Environment, University of Chinese Academy of Sciences, Beijing 100049, China

**Keywords:** radiometric capability, reflective bands, CCD, VIRR, MERSI, radiometric stability, radiometric accuracy

## Abstract

The radiometric capability of on-orbit sensors should be updated on time due to changes induced by space environmental factors and instrument aging. Some sensors, such as Moderate Resolution Imaging Spectroradiometer (MODIS), have onboard calibrators, which enable real-time calibration. However, most Chinese remote sensing satellite sensors lack onboard calibrators. Their radiometric calibrations have been updated once a year based on a vicarious calibration procedure, which has affected the applications of the data. Therefore, a full evaluation of the sensors’ radiometric capabilities is essential before quantitative applications can be made. In this study, a comprehensive procedure for evaluating the radiometric capability of several Chinese optical satellite sensors is proposed. In this procedure, long-term radiometric stability and radiometric accuracy are the two major indicators for radiometric evaluation. The radiometric temporal stability is analyzed by the tendency of long-term top-of-atmosphere (TOA) reflectance variation; the radiometric accuracy is determined by comparison with the TOA reflectance from MODIS after spectrally matching. Three Chinese sensors including the Charge-Coupled Device (CCD) camera onboard Huan Jing 1 satellite (HJ-1), as well as the Visible and Infrared Radiometer (VIRR) and Medium-Resolution Spectral Imager (MERSI) onboard the Feng Yun 3 satellite (FY-3) are evaluated in reflective bands based on this procedure. The results are reasonable, and thus can provide reliable reference for the sensors’ application, and as such will promote the development of Chinese satellite data.

## 1. Introduction

The stability and consistency of satellite sensors are critical for providing consistent data and products for the monitoring of Earth’s resources and dynamics [1]. Remote sensing imagery is an effective tool for monitoring global environment and climate changes because of its long-term observations [2]. The quantitative applications using remote sensing are sensitive to the sensor’s radiometric capability [3]. Discerning secular trends in geophysical processes is one goal of earth observation research, thus long-term data from remote sensing instruments with good performance is required [4].

Frequent calibration for on-orbit sensors is required. As Wyatt [5] pointed out, radiometric calibration is a key radiometric characteristic required for understanding an instrument’s performance. Sensors’ radiometric capability can be expected to change during launch and on-orbit operations because of the space environmental varying and instrument aging [6]. In order to correct the changes, regular and reliable onboard absolute calibration are required to assure a sensor’s radiometric capability with good accuracy and stability. For those satellite sensors with a spaceborne calibration system, like Moderate Resolution Imaging Spectroradiometer (MODIS), spectral response shifts and bandwidth changes on-orbit can be tracked by an onboard calibrator. For some satellite sensors without onboard calibration calibrators, such as Advanced Very-High-Resolution Radiometer (AVHRR), they use vicarious calibration techniques once a month to provide full aperture calibrations with relatively high accuracy as an independent evaluation of the sensor performance [7].

However, most Chinese remote sensing satellite sensors lack onboard calibrators and radiometric calibration has been updated once a year based on a vicarious calibration procedure, which has a great influence on the application of the data. Since the polar orbiting meteorological satellite, the Feng Yun 1 (FY-1, the first satellite in FY series) launched on 9 July 1988, which signifies an unprecedented milestone in Chinese satellite remote sensing history, several series of Chinese optical satellites have been developed, like the China Brazil Earth Resource Satellite (CBERS), Huan Jing (HJ), Zi Yuan (ZY), Hai Yang (HY), and Gao Fen (GF). The FY series could effectively monitor typhoons, floods, forest and grassland fires, droughts, dust storms and other disasters, improve the ability of weather forecasting and climate change detection [8]. The HY series has been used to monitor the sea ice, sea surface temperature and wind field and predict catastrophic sea-states such as storm, tsunami, sea-ice, sea-fog and marine petroleum pollution [9]. The ZY-3 series is mainly used in cartography, DEM modeling and resources investigation [10]. The HJ series satellites provide important support for detection of surface water quality and atmospheric environment, and evaluation of environmental pollution or natural disasters [11]. In general, due to the continuous development of aerospace remote sensing, satellites have been successfully and widely applied in the fields of agriculture, forestry, land, water conservancy, urban and rural construction, environment, surveying and mapping, transportation, meteorology, ocean, earth science research, and so on. The continuity and consistency of Earth’s surface measurements through time is the most crucial aspect in monitoring activities using these Earth-observing satellites. Due to the environmental factors of space and instruments aging, many of the changes will become apparent after decades or more of observation, so the sensors’ attenuation should be updated in a timely fashion. However, unlike the MODIS, most Chinese remote sensing satellite sensors lack onboard calibrators. Radiometric updates through a vicarious calibration procedure occur once a year, as for the HJ and FY series, which greatly influences the application of data. Therefore, a comprehensive evaluation of the sensors’ radiometric capability is essential before quantitative applications can take place.

The objective of this study is to propose a comprehensive procedure for evaluating the radiometric capability of Chinese optical satellite sensors and evaluate two major indicators for radiometric capability: the long-term radiometric stability and the radiometric accuracy. Three Chinese sensors including the Charge-Coupled Device (CCD) camera onboard the Huan Jing 1 satellite (HJ-1), Visible and Infrared Radiometer (VIRR) and Medium-Resolution Spectral Imager (MERSI) onboard the Feng Yun 3 satellite (FY-3) are evaluated in visible and infrared bands based on this procedure. The Badain Jaran Desert test site is selected because of its temporally stable surface condition, which minimizes the impacts of the surface and atmosphere variation. Long time series clear data (out of cloud and haze contamination) are selected, which guarantee a continuous and high frequency monitoring. The radiometric temporal stability is analyzed based on the tendency of long-term top-of-atmosphere (TOA) reflectance variation. The radiometric accuracy is determined by comparing with the TOA reflectance from MODIS after spectrally matching.

## 2. Materials and Methods

### 2.1. Test Site

**Figure 1:**
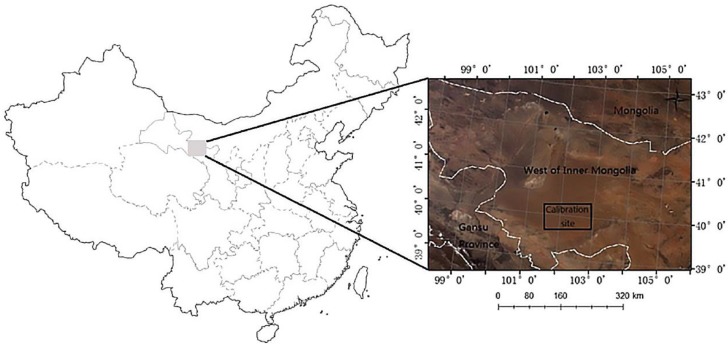
Location of the calibration site and a true color composite from Moderate Resolution Imaging Spectroradiometer (MODIS) imagery.

A site approximately of 30 km × 30 km located within the Badain Jaran Desert is adopted. It locates in central Inner Mongolia of Northern China (Figure 1). The site is selected for the following three reasons [12,13]: First, the area is temporally, spatially and radiationally stable in brightness, spatial homogeneity, altimetric and bidirectional effects, seasonal variation, and long-term stability [14]. Second, sand is the primary surface material in this area, provides a high-reflectance surface. Third, the site has a typical continental arid climate, and the atmosphere is always dry and clean (low aerosol).

### 2.2. Data

As a typical representative of moderate-high resolution remote sensing sensors, the CCD cameras are VNIR imaging sensors onboard the Chinese environment and disaster monitoring and forecasting satellite constellation, which is called Huan Jing 1 in Chinese and abbreviated as HJ-1 (hereafter the CCD camera onboard HJ-1 satellite is written as HJ-1/CCD). The HJ shoulders heavy responsibility of environmental monitoring, disaster prevention and reduction in China, and has been in orbit for more than six years and remains in operation, despite a two-year design life. However, due to lack of an onboard calibration system to track HJ-1 sensors’ on-orbit behavior throughout the life of the mission, vicarious calibration needs to be done every other time and the frequency is usually once a year and will be increased with the aging of instruments. Since the CCD cameras are not state-of-the-art instruments, the radiometric capability is not so stable that it does not change for the whole year. In order to obtain HJ-1/CCD data which are accurately and continuously calibrated, CRESDA performs the vicarious calibration measurements using the reflectance-based method at the Dunhuang calibration site, and releases the calibration coefficients once a year through its website at http://www.cresda.com.

The Feng Yun-3 satellite constellation, the second generation of Chinese polar-orbiting meteorological satellites with moderate-low resolution, is composed of three satellites: FY-3A, FY-3B, and FY-3C, respectively. FY-3A and FY-3C were launched on 27 May 2008 and 23 September 2013 by China National Satellite Meteorological Center (NASC), separately, in a sun-synchronous morning orbit with a local equator-crossing time of 10:30 a.m. in descending node, while FY-3B was launched on 5 November 2010 by NASC, in an afternoon orbit with an equator-crossing time of 1:30 p.m. in ascending node [15]. The MERSI and VIRR are the keystone payloads onboard FY-3. They are designed to enhance Chinese three-dimensional atmospheric sounding capability and global data acquisition capability. The MERSI images the Earth with 20 spectral bands covering from visible to long-wave infrared. The applications of MERSI include retrieval of true color image; monitoring cloud, ice, and snow coverage; monitoring ocean color; and detecting disasters such as flooding and wildfires [16]. The VIRR is a 10-channel VIS/IR multi-purpose imaging radiometer, with a spectral range from 0.43 μm to 12.5 μm. VIRR is the only instrument on the current FY-3 platform that can provide high spatial resolution and narrow-band infrared window measurements, which are vital for cloud or fog detection and quantitative retrieval of cloud properties, surface temperature, and the earth radiation budget [17]. Annual field campaigns have been routinely carried out at China Radiometric Calibration Site (CRCS) since September 2008, and calibration coefficients is released or updated through its website at http://www. nsmc.cma.gov.cn [18].

The MODIS sensor was launched on 18 December 1999 aboard the Terra satellite and has been in operation for nearly 17 years, providing global data that are used to monitor long-term changes in the Earth system. The absolute calibration of MODIS reflective solar bands is based on the regular measurements from the Solar Diffuser and a Solar Diffuser Stability Monitor [19,20,21]. Another onboard calibrator, the Spectroradiometric Calibration Assembly, is used to track the VNIR band’s spectral response shifts and bandwidth changes on-orbit [20]. MODIS also periodically views the moon through its space view port, which provides an additional point reference for the calibration [21]. The calibration uncertainties of the MODIS TOA reflectance products are ±2%, whereas a 5% uncertainty requirement is specified for the reflective solar bands at sensor spectral radiance calibration [22]. The MODIS instrument onboard the Earth Observing System’s (EOS) Terra and Aqua platforms have a state-of-the-art onboard calibration system with an absolute accuracy better than 2% [23], which has calibrated the solar reflective bands to reflectance units. Therefore, MODIS as a well-calibrated instrument is very practical for radiometric cross-calibrating those sensors without a good onboard calibration system by using targets of known reflectance [24,25]. Furthermore, as one of the most stable sensors, MODIS has been widely used for radiometric capability evaluation of other sensors [6,11,25]. In this paper, the MODIS level 1b data including MOD02 (Level-1B Calibrated Geolocation Data Set) and MOD03 (Geolocation Data Set is chosen as the reference data to evaluate the radiometric capability of the three sensors.

The primary characteristics of the CCDs, MERSIs, VIRRs and MODIS are listed in Table 1.

### 2.3. Methods

**Figure 2:**
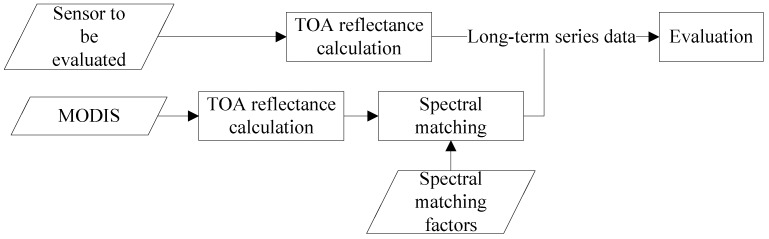
The block diagram of the framework for radiometric capability evaluation.

The procedure in this paper for evaluating radiometric capability of Chinese optical satellite sensors using MODIS is shown in Figure 2 and it includes three parts: (1) calculating the TOA reflectance; (2) spectral matching to eliminate the influence of different spectral response; (3) evaluating the radiometric capability of the sensors using the long-term series TOA reflectance between sensors and MODIS after spectral matching.

#### 2.3.1. Top-of-Atmosphere (TOA) Reflectance Calculation

The HJ-1/CCD data used in this study are derived from the Level 2 products, and the ground observation station of Chinese resources satellites has conducted radiation correction on these data to correct for the radiation deviation of the sensors. The TOA reflectance of the CCD can be calculated using Equation (1).

(1) $$\rho_{\lambda }=\frac{\pi L_{\lambda }d^{2}}{ESUN_{\lambda }\cdot \sin(\theta_{SE})}$$

where $\rho_{\lambda }$ is the TOA reflectance; $L_{\lambda }$ is the TOA radiance; d is the Earth-Sun distance in astronomical units; $\theta_{SE}$ is the solar elevation; $ESUN_{\lambda }$ is the solar irradiance at the top of the atmosphere; and $L_{\lambda }$ can be calculated using Equation (2)

(2) L=DN/gain+offset

Table 2 presents the gains and offsets of HJ-1/CCDs. Of those, the gains of 2008 are obtained by prelaunch Lab calibration, and the gains of 2009–2012 are obtained by post launch site calibration.

The TOA reflectance of the FY-3A/MERSI can be calculated using Equation (3).

(3) $$\rho_{\lambda }=\frac{(k_{0}+k_{1}\cdot DN+k_{2}\cdot DN^{2})\cdot d^{2}}{100\cdot \cos(\theta_{SZ})}$$

where $k_{0}$, $k_{1}$ and $k_{2}$ is the calibration coefficients, which can be read from the data source file. For the record, the calibration coefficients are not always the same because the vicarious calibration is routinely carried out annually, and updated through the China National Satellite Meteorological Center (NSMC) website (http://www.nsmc.cma.gov.cn/). The calibration coefficients of FY-3A/MERSI published by NSMC have experienced three updates since the launch. That is, four stages of calibration can be divided from launch until 2015. All the calibration coefficients are listed in Table 3.

The radiometric calibration of the FY-3B/MERSI has experienced two stages. In the first stage, the TOA reflectance of the FY-3B/MERSI data from 18 October 2010 to 6 March 2013 can be calculated using Equation (4) using coefficients in Table 4. After that, the organization structure of the FY-3B/MERSI data source file has been updated and calibration equation is changed. The TOA reflectance of the FY-3B/MERSI data from 6 March 2013 until now can be calculated using Equation (4)

(4) $$\rho_{\lambda }=\frac{RefFactor\cdot d^{2}}{100\cdot \cos(\theta_{SZ})}$$

where RefFactor is the factor of reflectance, and can be read from the data source file.

Both FY-3A/VIRR and FY-3B/VIRR data can be calibrated using Equation (5)

(5) $$\rho_{\lambda }=\frac{(S\cdot C_{E}+I)\cdot d^{2}}{100\cdot \cos(\theta_{SZ})}$$

where S is the calibration slope, I is the calibration intercept, and $C_{E}$ is the channel count value. S, I and $C_{E}$ are calibration coefficients and can be obtained from the data source file. Both of them have two stages of calibration coefficients, which are listed in Table 5.

Unlike the CCDs, MERSIs and VIRRs, whose calibrations are performed relying on the site calibration annually, the calibration of MODIS in VNIR bands are based on the regular measurements from the onboard calibrators, so the calibration coefficients of MODIS data are different according to the data acquisition time and satellite attitude, etc. The TOA reflectance of the MODIS can be calculated using Equation (6).

(6) $$\rho_{\lambda }=reflectance\_scale\cdot (DN-reflectance\_offsets)/\cos(\theta_{SZ})$$

where $\rho_{\lambda }$ is the TOA reflectance; $\theta_{SZ}$ is the solar zenith angle; reflectance_scale and reflectance_offsets are the gain and offset of the reflectance, respectively; DN is the channel count value.

#### 2.3.2. Spectral Matching

**Figure 3:**
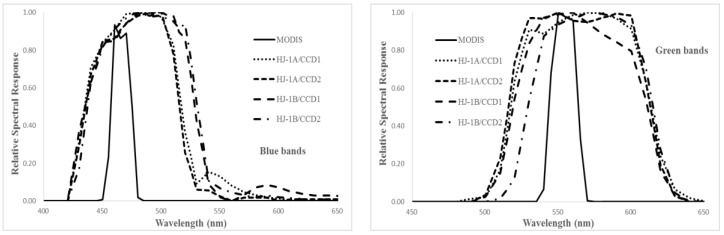

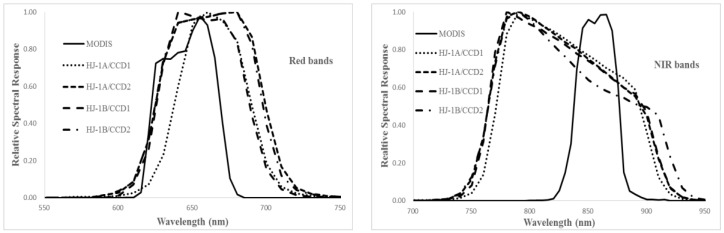
The relative spectral responses of the CCDs and MODIS in VNIR bands.

**Figure 4:**
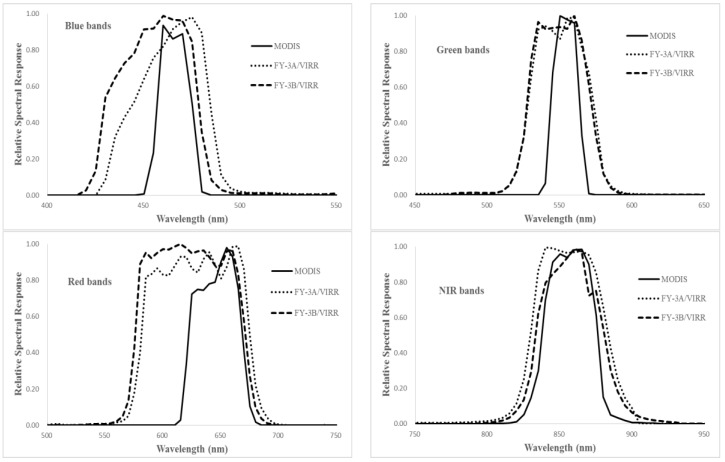
The relative spectral responses of the VIRRs and MODIS in VNIR bands.

**Figure 5:**
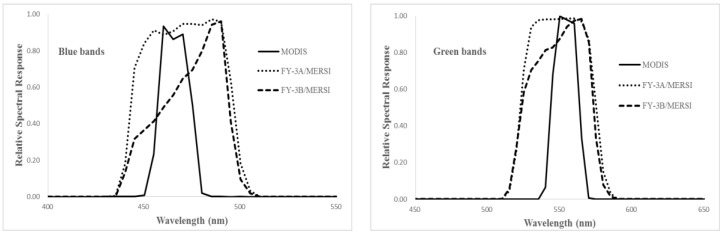

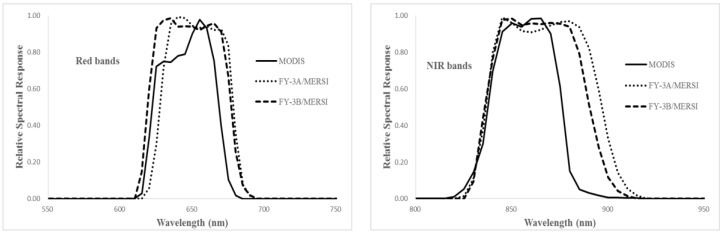
The relative spectral responses of the MERSIs and MODISs in VNIR bands.

Figure 3, Figure 4 and Figure 5 show the relative spectral responses of the CCDs, MERSIs, VIRRs and MODISs in blue, green, red, and NIR bands. These relative spectral response profiles differ in shape, bandwidth, peak value, location of the central wavelength, and degree of overlap between channels. Since the similar principle of design, the relative spectral response profiles of the sensors are almost unanimous.

Due to the atmospheric transmittance and the atmosphere outside solar irradiance varying with wavelength, different responses will affect the sensors’ reflectances at the top of the atmosphere. Thus, spectral matching is performed to compare radiation characteristics between the sensors to be evaluated and MODIS. Given the spectral behavior of the site (the ground feature is desert) and the spectral response functions of the instruments, the spectral difference between MODIS and other sensors can be expressed as a linear translation equation of the form $\rho_{sensor,i}=a_{i}\cdot \rho_{modis,j}$, where i represents band number of the other sensor and j is the corresponding band number of MODIS. The spectral difference coefficient $a_{i}$ is calculated based on the Equation (7) [14,26,27].

(7) $$a=\int_{\lambda_{1}}^{\lambda_{2}}\rho_{\lambda }\times f_{sensor}(\lambda )d\lambda /\int_{\lambda_{3}}^{\lambda_{4}}\rho_{\lambda }\times f_{\mathrm{mod}is}(\lambda )d\lambda$$

where $f_{sensor}(\lambda )$ and $f_{modis}(\lambda )$ are the relative spectral response functions for other sensors and MODIS, respectively. $\lambda_{1}$−$\lambda_{2}$ is the spectral range of other sensor; $\lambda_{3}$−$\lambda_{4}$ is the spectral range of MODIS. The ground-measured spectrum of the calibration site we used in this paper comes from the measurement in the Badain Jaran Desert [14]. Based on the definition of the spectral matching factor, the spectral matching factors between the sensors and the MODIS in reflective bands are calculated and listed in Table 6.

#### 2.3.3. Evaluation Method

Radiometric stability and accuracy are the two most important indicators to evaluate a sensor’s radiometric capability. Radiometric stability is defined as follows: given a calibration site with very stable radiometric characterizations, such as Badain Jaran Desert in this study, the radiance measured by the sensor to be evaluated is stable temporally. Ideally, it would be permanent, but it is usually defined as the sensor’s lifetime. In this study, the radiometric stability of a sensor is analyzed by using the tendency of long-term top-of-atmosphere (TOA) reflectance at the calibration site. Radiometric accuracy is defined as how the radiance measured from the sensor is close to the actual radiance measured using ground instruments with high precision. In this study, the MODIS is used as the standard instruments with excellent performance on radiometric capability and the radiometric accuracy of a sensor is determined by comparing with the TOA reflectance from MODIS after spectrally matching.

In order to evaluate the radiometric stability and accuracy for a given sensor, a procedure including three steps is worked out and the procedure is as follows:

Firstly, the long-term TOA reflectance of MODIS is plotted and the stability of MODIS is analyzed. Furthermore, it can be compared to the to-be-evaluated Chinese sensors visually.

Secondly, the long-term TOA reflectance of each reflective band for every sensor is plotted, which can illustrate the radiometric stability very intuitively. If the stability is good enough, a linear fitting of the long-term radiometric tendency can be done and the stability can be quantitatively described using the slope of the fitted line. If the stability is not good, the possible reasons can be analyzed based on the plot.

Thirdly, compare the TOA reflectance between the sensor to be evaluated and the MODIS to get the radiometric accuracy of the to-be-evaluated sensors. By employing some indices including maximum, minimum, mean, and standard deviation of TOA reflectance, the accuracy of the sensor can be quantified.

The indices for evaluating a sensor’s radiometric capability are summarized as follows:

(1) Slope: the slope of the fitted line for long-term tendency of the TOA reflectance at the calibration site indicates the radiometric stability of a sensor. When a slope value is close to 0, it means the TOA reflectance remains stable. A positive slope means an increasing tendency of TOA reflectance and a negative one means a decreasing tendency.
(2) Maximum and minimum values: the maximum value and minimum value indicate the largest and smallest TOA reflectance within the long time TOA reflectance, respectively. If the maximum value is within the proper range, it is usually induced by some self-factors, such as intra-annual variation, observation angles, solar angles, aerosols, and so on; however, if the maximum value is out of the proper range, it indicates that some external factors are working.
(3) Mean value: the mean value indicates the average of the TOA reflectance.
(4) Standard deviation. The standard deviation is a measure that is used to quantify the amount of variation or dispersion of a set. A low standard deviation indicates that the elements tend to be close to the mean of the set, while a high standard deviation indicates that the elements are spread out over a wider range of values. The standard deviation can be calculated using the Equation (8):
  (8) $$\sigma =\sqrt{\frac{1}{N}\sum_{i=1}^{N}\rho_{abs(i)}-\bar{\rho_{abs}}}$$
  where i represents serial number of the selected images, $\bar{\rho_{abs}}$ is the mean value of the TOA reflectance, and $\rho_{abs(i)}$ is the value of the TOA reflectance difference.

## 3. Results and Discussion

### 3.1. Radiometric Capability Evaluation of Moderate Resolution Imaging Spectroradiometer (MODIS)

**Figure 6:**
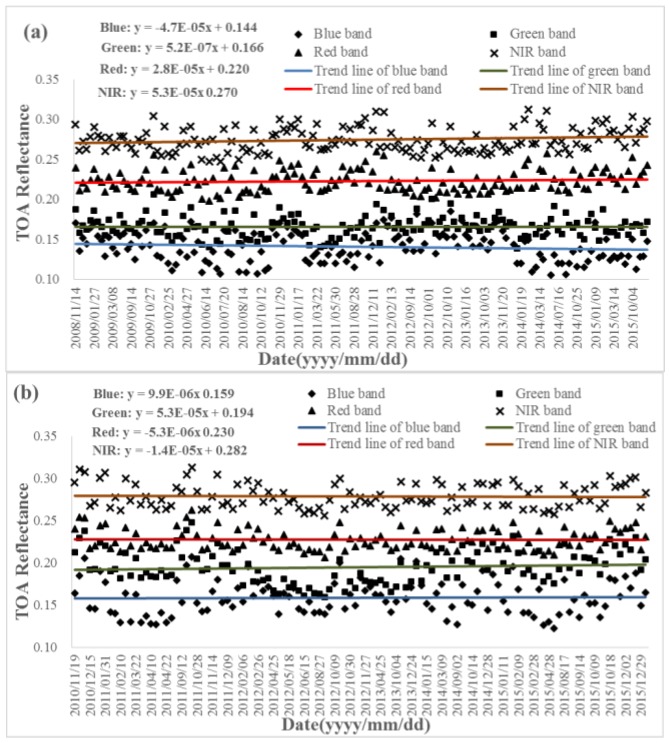
Time series of TOA reflectance of MODIS in reflective bands. The color lines are the trend lines of the bands. (**a**) Terra/MODIS; (**b**) Aqua/MODIS.

The Terra/MODIS acquired from 2008 to 2015 and the Aqua/MODIS acquired from 2010 to 2015 have been collected in order to evaluate the temporal radiometric capability of the two sensors. Figure 6 shows that the stability of MODIS from both Terra and Aqua are visually excellent. Subsequently, in order to evaluate the radiometric capability of MODIS quantitatively, a linear fitting to the TOA reflectance data was carried out and all the statistics are listed in Table 7. Table 7 summarizes the slopes and intercepts of the trend lines, and the maximum, minimum, mean and standard deviation values of the TOA reflectances in reflective bands including blue, green, red, and NIR. Several things about the table need to be clarified.

**Figure 7:**
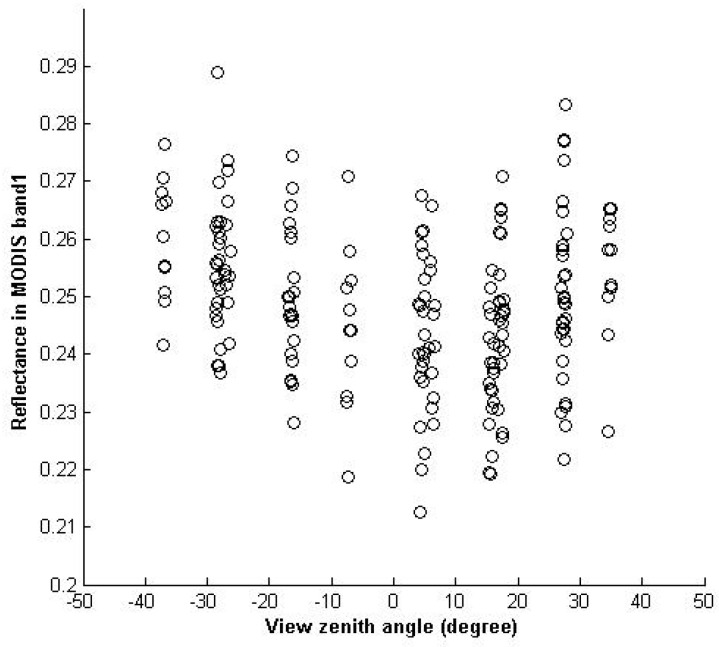
The calibration site’s directional characterization of the red band of Terra/MODIS. The reflectance varies with relative azimuth and solar zenith angle for each bin of view zenith angle, which shows systematic variation that is due to directional effects. The directional effect is about 15%.

(1) The difference between maximum and minimum values indicates the range of TOA reflectance variation. The blue band usually has a larger variation than the other three bands and the variation becomes smaller and smaller with an increasing wavelength, because the TOA reflectance at a shorter wavelength incorporates more scattering radiation from the atmosphere, and the atmospheric effect becomes stronger with the increasing of aerosols. For example, the difference of TOA reflectance at the blue band for Terra is 0.185 − 0.106 = 0.079; compared to the mean TOA reflectance of 0.14, the variation reaches 0.079/0.14 = 56.43%. The variations of the other three bands are 40.96%, 27.80%, and 23.72% respectively. In addition, the minimum, maximum, and the values close to the minimum and the maximum appear for most of the years, which clarifies that the maximum and minimum are not abnormal values but rather the normal ones, and that the aerosol condition varies day by day, though the variation is very similar year by year.
(2) Besides the atmospheric effect, the BRDF also contributes to the variation of the TOA reflectance. Figure 7 gives an example of the directional effect of the red band of Terra/MODIS. The reflectance varies with relative azimuth and solar zenith angle for each bin of view zenith angle, which shows systematic variation that is due to directional effects. The directional effect is about 15%.
(3) The mean of TOA reflectance at the same band are usually a little bit different, especially at the blue and red bands. The radiometric accuracy of Aqua is slightly better than that of Terra [28].

Although several factors can induce the variation of the TOA reflectance, the long-term tendency of the TOA reflectance remains consistent and the slope values of the fitted lines for different bands range from 10^−7^ to 10^−5^, which indicates a very small variation trending. The intercepts of the fitted lines are close to the corresponding means of TOA reflectance. The standard deviations of the TOA reflectance are within 0.02. All of the above show that the radiometric capability of both Terra/MODIS and the Aqua/MODIS are stable, and can be used to evaluate other sensors as the reference data.

### 3.2. Radiometric Capability Evaluation of Huan Jing 1/Charge-Coupled Device (HJ-1/CCD)

**Figure 8:**
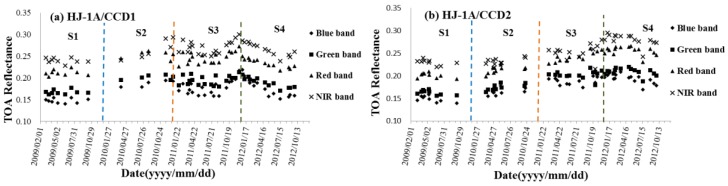

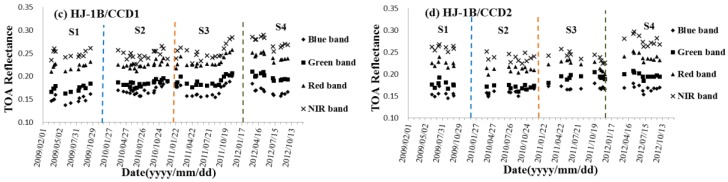
Time series of TOA reflectance of HJ-1/CCDs from 2008 to 2012 in reflective bands: (**a**) HJ-1A/CCD1; (**b**) HJ-1A/CCD2; (**c**) HJ-1B/CCD1; (**d**) HJ-1B/CCD2.

The same plots of the HJ-1/CCDs as the MODISs are presented in Figure 8. The temporal range of HJ-1/CCDs is 2009~2012 when the sensors are operational and in excellent condition. In Figure 8, it can be observed that the stability of the four CCDs is not as good as the MODIS for pronounced calibration drifts. The TOA reflectance of HJ-1/CCD varies periodically and the turning points are exactly the same as the calibration time, which is done once a year based on a vicarious calibration, thereby demonstrating that the procedure of the calibration may have some issues. However, as we have not involved the calibration, this is just an assumption.

Table 8, Table 9, Table 10 and Table 11 summarize the statistics of the CCDs; the radiometric characteristics are as follows:

(1) For HJ-1A/CCD1 (Table 8), the TOA reflectance in 2009 is the most stable, especially the blue band with a slope at the 10^−7^ level. A gradual decreasing tendency is observed in 2012 for all bands (slopes all less than 0). The TOA reflectance tendency in 2011 in blue and NIR bands do not agree with the linear rule; consequently, their slopes are not calculated. The highest variation of TOA reflectance occurs in 2011 in blue band, up to (0.2193 − 0.1585)/0.1795 = 33.87%, while the lowest occurs in 2010 in the green band: 6.32%. The mean TOA reflectances of HJ-1A/CCD1 after spectral matching are all higher than that of MODIS in the blue and green bands from 2009–2012, while lower than that of MODIS in the NIR bands of the four years. All the standard deviations of the TOA reflectance are within 0.02, and the lowest standard deviations occur in 2009 (0.0054 for blue band, 0.0052 for green band, 0.0067 for red band, and 0.0062 for NIR band, respectively).
(2) For HJ-1A/CCD2 (Table 9), the TOA reflectance in 2011 is the most stable, especially the blue band with a slope at the 10^−6^ level. A gradual decreasing tendency is observed in 2009 and 2012 for all bands, while the increasing tendency is observed in 2010 and 2011 for all bands. The highest variation of TOA reflectance occurs in 2011 in the NIR band, up to 25.30%, while the lowest occurs in 2009 in the red band: 7.91%. Blue bands of HJ-1A/CCD2 in 2009, 2010 and 2012 have the largest variation over the other three bands. The mean TOA reflectances of HJ-1A/CCD2 after spectral matching are higher than those of MODIS except the red and NIR bands in 2009 and 2010, and the NIR band in 2011. All the standard deviations of the TOA reflectances are within 0.02, and the lowest standard deviations occur in 2009 (0.0069 for blue band, 0.0052 for green band, 0.0057 for red band, and 0.0063 for NIR band, respectively).
(3) For HJ-1B/CCD1 (Table 10), the TOA reflectance in 2009 is the most stable due to the slopes in the four bands being smaller than the other three years. A gradual decreasing tendency is observed in 2012 for all bands, while the increasing tendency is observed in 2009 for all bands. The TOA reflectance tendency in 2010 in the blue band and 2011 in all the four bands do not agree with linear rule; consequently, their slopes are not calculated. The highest variation of TOA reflectance occurs in 2011 in the blue band, up to 26.94%, while the lowest occurs in 2012 in the green band: 9.76%. Blue bands of HJ-1B/CCD1 have the largest variation over the other three bands. The mean TOA reflectances after spectral matching are higher than those of MODIS, except the NIR bands in 2009, 2010, and 2011. All the standard deviations of the TOA reflectance are within 0.02, and the lowest standard deviations occur in 2009 (0.0072 for blue band, 0.0066 for green band, 0.0070 for red band, and 0.0084 for NIR band, respectively).
(4) For HJ-1B/CCD2 (Table 11), the TOA reflectance in 2009 is the most stable. A gradual decreasing tendency is observed in 2014 for all bands. The TOA reflectances’ tendency in 2011 in red and NIR bands do not agree with linear rule and their slopes are not calculated. The highest variation of TOA reflectance occurs in 2009 in the eblue band, up to 17.63%, while the lowest occurs in 2009 in the NIR band: 6.35%. Blue bands of HJ-1B/CCD1 have the largest variation of the four bands except in 2011. The mean TOA reflectances after spectral matching are higher than those of MODIS except the NIR bands in 2009, 2010, and 2011, and red band in 2010. All the standard deviations of the TOA reflectances are within 0.02, and the lowest standard deviations in blue, green, red and NIR bands occur in 2011 (0.0044), 2012 (0.0061), 2010 (0.0056) and 2009 (0.0055), respectively.
(5) Both the TOA reflectances in blue and green bands of the four CCDs are higher than those of the MODIS, while the TOA reflectance in the red band is much closer to that of the MODIS. However, almost all the TOA reflectances in NIR bands are lower than those of the MODIS.
(6) Compared to the HJ-1A/CCDs, the HJ-1B/CCDs are more stable because almost all the slopes of the TOA reflectance tendency of HJ-1B/CCDs are lower than those of the HJ-1A/CCDs in the corresponding year and band.
(7) At the beginning of the operation of an instrument, the vicarious calibration once a year is maybe enough to ensure the radiometric capability; however, it cannot guarantee the radiometric capability after the beginning stage of the operation. Subsequently, some new and highly frequent calibration methods need to be proposed and executed by the instruments’ surveillance department.

### 3.3. Radiometric Capability Evaluation of Feng Yun 3/Medium-Resolution Spectral Imager (FY-3/MERSI)

**Figure 9:**
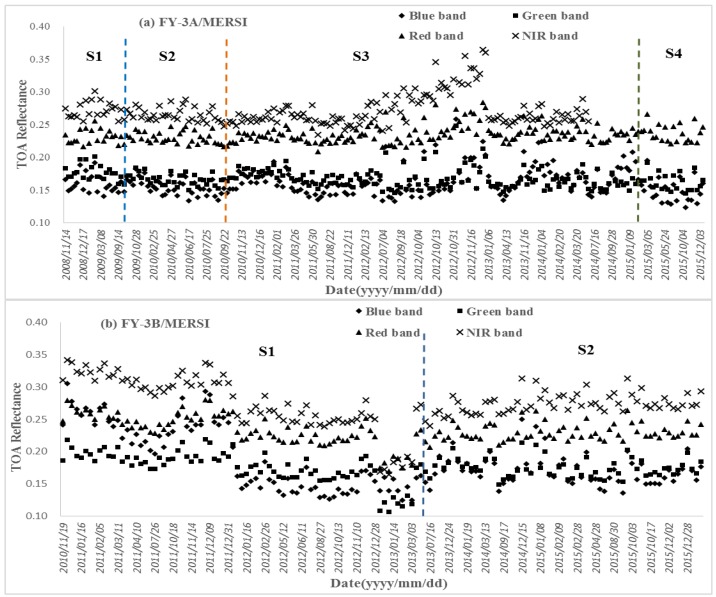
Time series of TOA reflectance of MERSIs in reflective bands: (**a**) FY-3A/MERSI from 2008 to 2015; (**b**) FY-3B/MERSI from 2010 to 2015.

The same plots of the FY-3/MERSIs as the MODISs are presented in Figure 9. The temporal range of FY-3A/MERSI is 2008~2015 and that of FY-3B/MERSI is 2010~2015. The radiometric characteristics of the MERSIs are as follows:

(1) The stability of FY-3A/MERSI is very good at all times except the duration from February to December of 2012, when a strongly increasing trend occurred. Although calibration coefficients have been updated four times, the variation of the TOA reflectance remains consistent. The reasons inducing the abnormal increasing within stage 3 is unknown and we surmise that it may be caused by an instrument malfunction. After it was found by the surveillance department, a manual adjustment was made and the instrument suddenly became normal. The statistics for FY-3A/MERSI are calculated, excluding the instrument malfunction duration, and listed in Table 12. The slope values of the fitted lines for different bands are at the 10^−5^ level, which indicates a very small variation trending. The highest variation of TOA reflectance occurs in the blue band, up to 62.72%. The variations of the other three bands are 33.74%, 24.86%, and 25.52% respectively. The differences in four bands between the mean TOA reflectance after spectral matching and corresponding bands of MODIS are all less than 0.015. All the standard deviations of the TOA reflectance are within 0.02. After the malfunction duration is removed, the stability and accuracy are very close to that of MODIS.
(2) The stability of FY-3B/MERSI has a two-stage mode. At the first stage, three sub-stages are very obvious. An obvious over-estimation of the TOA reflectance appears at the first sub-stage; the second sub-stage has a close estimation; the third sub-stage has an under-estimation. The three sub-stages changed suddenly, so manual adjustments of the instrument were executed by the surveillance department. However, the radiometric stability and accuracy have become better and very close to the MODIS’s. Only the statistics of the second stage are calculated and listed in Table 12. The TOA reflectance in green and red bands has a tendency towards lightly decreasing because all the slopes are at the 10^−4^ level and greater than 0. The highest variation of TOA reflectance occurs in the blue band, up to 41.75%. The variations of the other three bands are 28.52%, 20.75%, and 18.39% respectively. The differences in four bands between the mean TOA reflectance after spectral matching and corresponding bands of MODIS are less than 0.015 except the green band (0.023). All the standard deviations of the TOA reflectance are within 0.02. The stability and accuracy of FY-3B/MERSI in the second stage are very good.
(3) Although both the two MERSIs have very low frequency of calibration, the stability remains very good; however, with the degradation of the instruments after a long operation, it is difficult to retain very good radiometric capability with very low frequent calibrations. Moreover, high frequent calibrations can find the instruments’ malfunction timely.

### 3.4. Radiometric Capability Evaluation of FY-3/Visible and Infrared Radiometer (VIRR)

**Figure 10:**
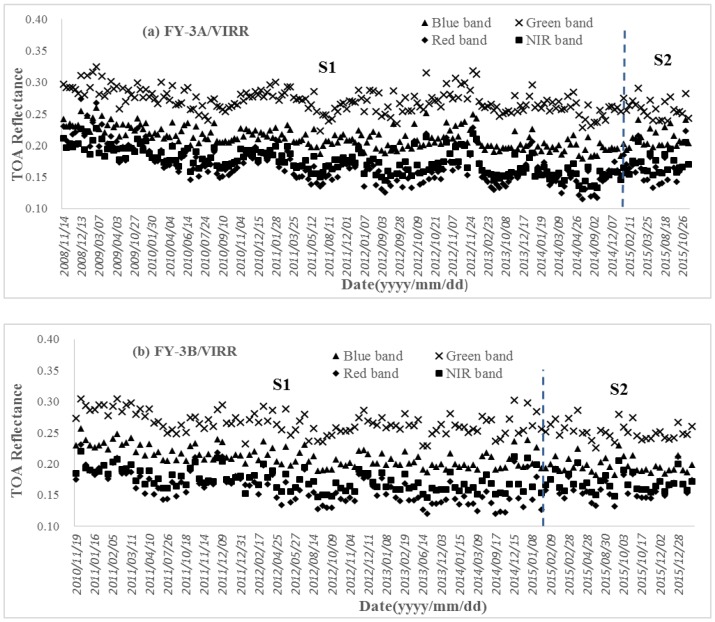
Time series of TOA reflectance of VIRRs in reflective bands: (**a**) FY-3A/VIRR from 2008 to 2015; (**b**) FY-3B/VIRR from 2010 to 2015. The color lines separate different calibration stages.

The same plots of the FY-3/VIRRs as the MODISs are presented in Figure 10. The temporal range of FY-3A/VIRR is 2008~2015 and that of FY-3B/VIRR is 2010~2015. The radiometric characteristics of the VIRRs are as follows:

(1) Both VIRRs have a tendency toward obvious decreasing in their first calibration stages. The reason for the trend can probably be attributed to the degradation of the instruments. At the beginning of the operations of VIRRs, the TOA reflectance in the four bands are abnormally high, especially in the blue band and even higher in the green band. After further investigated and constant adjustment, the instruments became normal and stable in the second stage. The statistics are calculated and listed in Table 13.
(2) For the FY-3A/VIRR, the variations of the four bands are 39.42%, 24.48%, 18.60%, and 15.01% respectively. The differences in four bands between the mean TOA reflectance after spectral matching and corresponding bands of MODIS are all less than 0.02. All the standard deviations of the TOA reflectance are within 0.02.
(3) For the FY-3B/VIRR, the variations of the four bands are 43.27%, 31.65%, 21.68%, and 23.90% respectively. Only the difference in blue band between the mean TOA reflectance after spectral matching and MODIS is close to 0 (0.0004). The mean TOA reflectance of the FY-3B/VIRR after spectral matching in other three bands are less than that of MODIS and the differences are higher than 0.02 (0.023 for green band, 0.022 for red band and 0.028 for NIR band). All the standard deviations of the TOA reflectance are within 0.02.
(4) Low frequency of calibrations is still a problem for VIRRS and a high frequent procedure for calibration could be a solution for the decreasing trend’s instability.

## 4. Conclusions

Since an excellent radiometric capability of satellite optical sensors is prerequisite, the radiometric capability needs to be evaluated first. Most of the new satellite optical sensors have onboard calibrators, which have the ability of real-time monitoring; therefore, an excellent radiometric capability can be expected. However, most of the Chinese satellite optical sensors lack onboard calibrators and the radiometric capability of these sensors needs to be evaluated often to ensure the quality of the data. Thus, this paper proposed an alternative procedure for monitoring the radiometric capability of Chinese optical satellite sensors using long time series data. In this procedure, two major indicators including radiometric stability and accuracy are employed for evaluation. Following an evaluation of the eight Chinese satellite optical sensors, the following conclusions can be made.

First of all, almost all of the Chinese satellite optical sensors are less stable than the Moderate Resolution Imaging Spectroradiometers (MODISs), and their radiometric accuracy is less than that of the MODISs.

Secondly, among all the evaluated sensors, the Visible and Infrared Radiometer (VIRRs) thus far have the best stability throughout their lifetime, although they have an obvious decreasing trend probably induced by instrument degradation; the Medium-Resolution Spectral Imagers (MERSIs) have the best radiometric performance in terms of both stability and accuracy at their later stages.

Thirdly, a vicarious calibration procedure carried out once a year or once every several years has been operated by the surveillance departments of the sensors, which has greatly contributed to improving the lower-quality radiometric capability of Chinese satellite optical sensors. However, a more frequent calibration procedure urgently needs to be developed and fulfilled in the future due to the lack of onboard calibrator. Moreover, in order to fully take advantage of the ample Chinese satellite data, the recalibration of the historical data also needs to be carried out in the future.

Fourthly, with the ongoing integrated global observations, more and more optical sensors onboard different satellites with global observing capability have been placed into orbit. Quantitative applications through multi-sensors require continued and consistent measurements from different instruments. This study provides reliable reference for the sensors’ applications, and as such will promote the development of Chinese satellite data.

In the near future, we will evaluate the radiometric capability of other Chinese optical satellite sensors, such as the Gao Fen (GF) and Zi Yuan (ZY) series’ satellites; subsequently, more abundant and reliable data from Chinese optical satellite sensors are expected, which will greatly contribute to research and applications.

## Figures and Tables

**Table 1:** The primary characteristics of participating bands of CCDs, MERSIs and VIRRs.

Satellite	Sensor	Band	Spectral Range (nm)	Spatial Resolution (m)	Revisit Period (Days)	Orbit Altitude (km)	Local Equator-Crossing Time	Field of View Angle (°)
HJ-1A/B	CCD1 and CCD2	Blue	430–520	30	4 days for one satellite; 2 days for A and B together	649	10:30 a.m. in descending node	31
		Green	520–600	30				
		Red	630–690	30				
		Near infrared	700–900	30				
FY-3	MERSI	Blue	445–495	250	5.5	836	FY-3A/MERSI: 10:30 a.m. in descending node FY-3B/MERSI: 1:30 a.m. in ascending node	55.4
		Green	525–575	250				
		Red	630–678	250				
		Near infrared	838–893	250				
FY-3	VIRR	Blue	431–490	1100	5.5	836	FY-3A/VIRR: 10:30 a.m. in descending node FY-3B/VIRR: 1:30 a.m. in ascending node	55.4
		Green	518–581	1100				
		Red	573–684	1100				
		Near infrared	820–898	1100				
Terra/Aqua	MODIS	Blue	459–479	500	5.5	705	Terra/MODIS: 10:30 a.m. in descending node Aqua/MODIS: 1:30 a.m. in ascending node	55.4
		Green	545–565	500				
		Red	620–670	250				
		Near infrared	841–876	250				

**Table 2:** The calibration coefficients of CCDs.

**HJ-1A/CCD1**								
**Years**	**Blue Band**		**Green Band**		**Red Band**		**NIR Band**	
	**Gain**	**Offset**	**Gain**	**Offset**	**Gain**	**Offset**	**Gain**	**Offset**
2008	0.916	7.325	0.9228	6.0737	1.1277	3.6123	1.0753	1.903
2009	0.6925	7.325	0.7438	6.0737	0.9636	3.6123	1.0545	1.903
2010	0.7768	7.325	0.7796	6.0737	1.0312	3.6123	1.0049	1.903
2011	0.7696	7.325	0.7815	6.0737	1.0914	3.6123	1.0281	1.903
2012	0.7069	7.325	0.7497	6.0737	1.0673	3.6123	1.0429	1.903
**HJ-1A/CCD2**								
**Years**	**Blue Band**		**Green Band**		**Red Band**		**NIR Band**	
	**Gain**	**Offset**	**Gain**	**Offset**	**Gain**	**Offset**	**Gain**	**Offset**
2008	0.9997	4.6344	1.0016	4.0982	1.3777	3.736	1.3043	0.739
2009	0.923	4.6344	0.9399	4.0982	1.3093	3.736	1.3178	0.739
2010	0.7892	4.6344	0.7831	4.0982	1.1635	3.736	1.1995	0.739
2011	0.7435	4.6344	0.7379	4.0982	1.0899	3.736	1.0852	0.739
2012	0.7257	4.6344	0.7291	4.0982	1.0464	3.736	1.0519	0.739
**HJ-1B/CCD1**								
**Years**	**Blue Band**		**Green Band**		**Red Band**		**NIR Band**	
	**Gain**	**Offset**	**Gain**	**Offset**	**Gain**	**Offset**	**Gain**	**Offset**
2008	0.8685	3.0089	0.9367	4.4487	1.2433	3.2144	1.3002	2.561
2009	0.7726	3.0089	0.8092	4.4487	1.117	3.2144	1.1337	2.561
2010	0.761	3.0089	0.7727	4.4487	1.0827	3.2144	1.1181	2.561
2011	0.706	3.0089	0.696	4.4487	1.0082	3.2144	1.0068	2.561
2012	0.6697	3.0089	0.7118	4.4487	1.0555	3.2144	1.1042	2.561
**HJ-1B/CCD2**								
**Years**	**Blue Band**		**Green Band**		**Red Band**		**NIR Band**	
	**Gain**	**Offset**	**Gain**	**Offset**	**Gain**	**Offset**	**Gain**	**Offset**
2008	0.9076	2.2219	0.8502	4.0683	1.1635	5.2537	0.98	6.35
2009	0.8394	2.2219	0.9006	4.0683	1.2461	5.2537	1.1261	6.35
2010	0.8352	2.2219	0.7925	4.0683	1.1316	5.2537	1.0578	6.35
2011	0.8042	2.2219	0.7822	4.0683	1.0556	5.2537	0.9237	6.35
2012	0.7587	2.2219	0.7629	4.0683	1.0245	5.2537	1.0146	6.35

**Table 3:** The calibration coefficients of FY-3A/MERSI.

**S1: 2008.11.11–2009.8.17**				**S3: 2010.8.20–2015.2.5**			
**Band**	**k2**	**k1**	**k0**	**Band**	**k2**	**k1**	**k0**
Blue	0.0000	0.0312	−7.5847	Blue	0.0000	0.0360	−8.3640
Green	0.0000	0.0295	−6.3543	Green	0.0000	0.0319	−6.8851
Red	0.0000	0.0253	−3.2586	Red	0.0000	0.0253	−3.2586
NIR	0.0000	0.0299	−3.6119	NIR	0.0000	0.0299	−3.6119
**S2: 2009.8.17–2010.8.20**				**S4: 2015.2.5–Current**			
**Band**	**k2**	**k1**	**k0**	**Band**	**k2**	**k1**	**k0**
Blue	0.0000	0.0339	−7.9511	Blue	0.0000	0.0406	−11.2490
Green	0.0000	0.0295	−6.3543	Green	0.0000	0.0319	−6.8851
Red	0.0000	0.0253	−3.2586	Red	0.0000	0.0253	−3.2586
NIR	0.0000	0.0299	−3.6119	NIR	0.0000	0.0299	−3.6119

**Table 4:** The calibration coefficients of FY-3B/MERSI.

S1: 2010.10.18–2013.3.6			
Band	k2	k1	k0
Blue	0.0000	0.0360	−8.3640
Green	0.0000	0.0319	−6.8851
Red	0.0000	0.0253	−3.2586
NIR	0.0000	0.0299	−3.6119

**Table 5:** The calibration coefficients of VIRRs.

**Sensor**	**S1: 2008.11.11–2015.2.5**			**Sensor**	**S1: 2010.10.18–2015.2.5**		
FY3A/VIRR	**Band**	**S**	**I**	FY3B/VIRR	**Band**	**S**	**I**
	Blue	0.0894	−1.1622		Blue	0.0748	−0.9098
	Green	0.0687	−0.8236		Green	0.0746	−0.8953
	Red	0.1457	−1.7484		Red	0.1264	−1.4320
	NIR	0.1435	−1.7348		NIR	0.1353	−1.6236
	**S2: 2015.2.5–Current**				**S2: 2015.2.5–Current**		
	**Band**	**S**	**I**		**Band**	**S**	**I**
	Blue	0.102	−1.3283		Blue	0.0938	−1.1494
	Green	0.08	−0.9576		Green	0.0803	−0.9636
	Red	0.1586	−1.892		Red	0.1264	−1.432
	NIR	0.1435	−1.7348		NIR	0.1353	−1.6236

**Table 6:** The spectral matching factors between CCDs, MERSIs, VIRRs and MODIS, respectively.

Sensor	a (Blue)	a (Green)	a (Red)	a (NIR)
HJ-1A/CCD1	0.8786	0.9065	0.9772	1.0002
HJ-1A/CCD2	0.9220	0.9186	0.9792	0.9990
HJ-1B/CCD1	0.8457	0.9193	0.9856	0.9988
HJ-1B/CCD2	0.8778	0.8749	0.9806	0.9988
FY3A/MERSI	1.0331	1.0486	1.0085	1.0016
FY3A/VIRR	0.9648	1.0009	0.9779	0.9808
FY3B/MERSI	1.0224	0.9775	1.0020	1.0003
FY3B/VIRR	1.0062	0.9791	0.9529	1.0009

**Table 7:** The statistics from the time series of the TOA reflectance of MODIS from both Terra and Aqua.

Sensor	Band	Slope	Intercept	Maximum	Minimum	Mean	Standard Deviation
Terra/MODIS	Blue	4.70 × 10^−5^	0.144	0.185	0.106	0.140	0.019
	Green	5.22 × 10^−7^	0.166	0.206	0.138	0.166	0.013
	Red	2.81 × 10^−5^	0.220	0.260	0.198	0.223	0.013
	NIR	5.33 × 10^−5^	0.270	0.312	0.247	0.274	0.015
Aqua/MODIS	Blue	9.87 × 10^−7^	0.159	0.207	0.122	0.159	0.020
	Green	5.30 × 10^−5^	0.194	0.248	0.160	0.195	0.020
	Red	−5.29 × 10^−6^	0.230	0.263	0.207	0.228	0.012
	NIR	−1.41 × 10^−5^	0.282	0.313	0.256	0.279	0.014

**Table 8:** The statistics from the time series of the TOA reflectance of HJ-1A/CCD1.

Year	Band	Slope	Maximum	Minimum	Mean	Mean_a *	Mean_a *-Mean_M ^#^	Standard Deviation
2009	Blue	2.67 × 10^−7^	0.1548	0.1408	0.1475	0.1679	0.0279	0.0054
	Green	8.88 × 10^−6^	0.1777	0.1621	0.1674	0.1847	0.0187	0.0052
	Red	7.04 × 10^−6^	0.2249	0.2032	0.2122	0.2171	−0.0059	0.0067
	NIR	1.16 × 10^−5^	0.2472	0.2280	0.2401	0.2400	−0.0340	0.0062
2010	Blue	5.76 × 10^−5^	0.1950	0.1792	0.1884	0.2144	0.0744	0.0073
	Green	1.73 × 10^−5^	0.2076	0.1949	0.2011	0.2218	0.0558	0.0053
	Red	−1.96 × 10^−6^	0.2629	0.2388	0.2533	0.2592	0.0362	0.0091
	NIR	2.34 × 10^−4^	0.2939	0.2407	0.2667	0.2666	−0.0074	0.0222
2011	Blue	-	0.2193	0.1585	0.1795	0.2043	0.0643	0.0184
	Green	2.98 × 10^−5^	0.2137	0.1805	0.1956	0.2158	0.0498	0.0109
	Red	5.56 × 10^−5^	0.2731	0.2264	0.2475	0.2533	0.0303	0.0129
	NIR	-	0.3006	0.2500	0.2717	0.2716	−0.0024	0.0149
2012	Blue	−2.04 × 10^−4^	0.2051	0.1516	0.1743	0.1984	0.0584	0.0188
	Green	−1.13 × 10^−4^	0.2024	0.1712	0.1879	0.2073	0.0413	0.0097
	Red	−8.56 × 10^−5^	0.2468	0.2167	0.2328	0.2382	0.0722	0.0097
	NIR	−1.06 × 10^−4^	0.2832	0.2386	0.2647	0.2646	−0.0094	0.0143

* Mean of the TOA reflectance after spectral matching with MODIS, and the same below. ^#^ Mean of the TOA reflectance of MODIS, and the same below.

**Table 9:** The statistics from the time series of the TOA reflectance of HJ-1A/CCD2.

Sensor	Year	Band	Slope	Maximum	Minimum	Mean	Mean_a	Mean_a *-Mean_M ^#^	Standard Deviation
HJ-1A/CCD2	2009	Blue	−6.73 × 10^−5^	0.1586	0.1392	0.1489	0.1695	0.0295	0.0069
		Green	−5.59 × 10^−5^	0.1700	0.1559	0.1629	0.1797	0.0137	0.0052
		Red	−4.26 × 10^−5^	0.2094	0.1935	0.2011	0.2058	−0.0172	0.0057
		NIR	−4.96 × 10^−5^	0.2399	0.2196	0.2300	0.2299	−0.0441	0.0063
	2010	Blue	3.56 × 10^−5^	0.1775	0.1552	0.1651	0.1879	0.0479	0.0071
		Green	6.67 × 10^−5^	0.1849	0.1637	0.1741	0.1920	0.0260	0.0071
		Red	4.89 × 10^−5^	0.2249	0.1984	0.2133	0.2183	−0.0047	0.0078
		NIR	6.77 × 10^−5^	0.2436	0.2165	0.2316	0.2315	−0.0425	0.0079
	2011	Blue	4.52 × 10^−5^	0.2171	0.1737	0.1909	0.2173	0.0773	0.0109
		Green	2.80 × 10^−5^	0.2178	0.1791	0.2026	0.2235	0.0593	0.0087
		Red	5.74 × 10^−5^	0.2606	0.2131	0.2409	0.2465	0.0235	0.0132
		NIR	5.23 × 10^−5^	0.2799	0.2154	0.2549	0.2548	−0.0192	0.0173
	2012	Blue	−7.56 × 10^−5^	0.2083	0.1694	0.1934	0.2201	0.0801	0.0107
		Green	−4.75 × 10^−5^	0.2195	0.1881	0.2095	0.2311	0.0651	0.0080
		Red	−3.59 × 10^−5^	0.2661	0.2307	0.2563	0.2623	0.0393	0.0096
		NIR	−7.90 × 10^−5^	0.2947	0.2422	0.2806	0.2805	0.0065	0.0130

**Table 10:** The statistics from the time series of the TOA reflectances of HJ-1B/CCD1.

Sensor	Year	Band	Slope	Maximum	Minimum	Mean	Mean_a	Mean_a *-Mean_M ^#^	Standard Deviation
HJ-1B/CCD1	2009	Blue	1.80 × 10^−5^	0.1614	0.1378	0.1499	0.1773	0.0373	0.0072
		Green	4.06 × 10^−5^	0.1842	0.1625	0.1730	0.1883	0.0223	0.0066
		Red	2.74 × 10^−5^	0.2314	0.2096	0.2208	0.2240	0.0010	0.0070
		NIR	1.16 × 10^−5^	0.2608	0.2353	0.2489	0.2492	−0.0248	0.0084
	2010	Blue	-	0.1874	0.1572	0.1694	0.2003	0.0603	0.0087
		Green	4.80 × 10^−5^	0.1954	0.1730	0.1848	0.2012	0.0352	0.0061
		Red	5.34 × 10^−5^	0.2400	0.2112	0.2248	0.2281	0.0051	0.0068
		NIR	3.88 × 10^−5^	0.2657	0.2258	0.2459	0.2462	−0.0278	0.0108
	2011	Blue	-	0.2004	0.1546	0.1700	0.2010	0.0610	0.0140
		Green	-	0.2069	0.1756	0.1889	0.2056	0.0396	0.0099
		Red	-	0.2572	0.2174	0.2321	0.2355	0.0125	0.0120
		NIR	-	0.2847	0.2281	0.2523	0.2526	−0.0214	0.0158
	2012	Blue	−7.65 × 10^−5^	0.2004	0.1576	0.1669	0.1974	0.0574	0.0075
		Green	−8.14 × 10^−5^	0.2097	0.1903	0.1988	0.2164	0.0504	0.0069
		Red	−8.48 × 10^−5^	0.2572	0.2293	0.2428	0.2464	0.0234	0.0076
		NIR	−1.25 × 10^−5^	0.2900	0.2537	0.2741	0.2744	0.0004	0.0113

**Table 11:** The statistics from the time series of the TOA reflectances of HJ-1B/CCD2.

Sensor	Year	Band	Slope	Maximum	Minimum	Mean	Mean_a	Mean_a *-Mean_M ^#^	Standard Deviation
HJ-1B/CCD2	2009	Blue	−2.91 × 10^−5^	0.1721	0.1449	0.1543	0.1758	0.0358	0.0081
		Green	−2.80 × 10^−5^	0.1918	0.1666	0.1763	0.2015	0.0355	0.0078
		Red	−4.75 × 10^−5^	0.2388	0.2144	0.2242	0.2286	0.0056	0.0075
		NIR	−1.70 × 10^−5^	0.2671	0.2506	0.2600	0.2607	−0.0133	0.0055
	2010	Blue	−4.66 × 10^−5^	0.1681	0.1486	0.1603	0.1826	0.0426	0.0064
		Green	−1.45 × 10^−5^	0.1753	0.1557	0.1687	0.1928	0.0268	0.0062
		Red	8.79 × 10^−6^	0.2168	0.1990	0.2100	0.2142	−0.0088	0.0056
		NIR	−1.99 × 10^−5^	0.2509	0.2244	0.2371	0.2377	−0.0363	0.0085
	2011	Blue	−2.23 × 10^−6^	0.1785	0.1642	0.1684	0.1918	0.0518	0.0044
		Green	1.44 × 10^−5^	0.2044	0.1801	0.1925	0.2200	0.0540	0.0062
		Red	-	0.2334	0.1994	0.2199	0.2243	0.0013	0.0113
		NIR	-	0.2569	0.2244	0.2393	0.2399	−0.0341	0.0101
	2012	Blue	−2.67 × 10^−5^	0.1789	0.1531	0.1647	0.1876	0.0476	0.0068
		Green	−5.43 × 10^−5^	0.2072	0.1844	0.1967	0.2248	0.0588	0.0061
		Red	−8.24 × 10^−5^	0.2519	0.2271	0.2387	0.2434	0.0204	0.0079
		NIR	−1.43 × 10^−5^	0.2964	0.2623	0.2768	0.2775	0.0035	0.0114

**Table 12:** The statistics from the time series of the TOA reflectance of FY-3/MERSIs.

Sensor	Band	Slope	Maximum	Minimum	Mean	Mean_a	Mean_a *-Mean_M ^#^	Standard Deviation
FY-3A/MERSI	Blue	2.04 × 10^−5^	0.2238	0.1235	0.1598	0.1547	0.0147	0.0179
	Green	−8.74 × 10^−5^	0.2012	0.1449	0.1670	0.1593	−0.0067	0.0115
	Red	4.62 × 10^−5^	0.2663	0.2084	0.2326	0.2306	0.0076	0.0103
	NIR	−3.58 × 10^−5^	0.3013	0.2342	0.2630	0.2626	−0.0114	0.0113
FY-3B/MERSI	Blue	8.80 × 10^−5^	0.2042	0.1354	0.1648	0.1708	0.0118	0.0174
	Green	2.77 × 10^−4^	0.2024	0.1534	0.1718	0.1716	−0.0234	0.0123
	Red	1.39 × 10^−4^	0.2638	0.2159	0.2308	0.2360	0.0080	0.0114
	NIR	−8.27 × 10^−5^	0.3136	0.2624	0.2784	0.2838	0.0048	0.0125

**Table 13:** The statistics from the time series of the TOA reflectance of FY-3/VIRRs.

Sensor	Band	Slope	Maximum	Minimum	Mean	Mean_a	Mean_a *-Mean_M ^#^	Standard Deviation
FY-3A/VIRR	Blue	−1.33 × 10^−6^	0.1951	0.1331	0.1573	0.1569	0.0169	0.0164
	Green	1.44 × 10^−4^	0.1939	0.1530	0.1671	0.1709	0.0049	0.0118
	Red	1.88 × 10^−4^	0.2353	0.1965	0.2086	0.2082	−0.0148	0.0109
	NIR	−7.06 × 10^−4^	0.2767	0.2385	0.2545	0.2544	−0.0196	0.0116
FY-3B/VIRR	Blue	−1.67 × 10^−4^	0.2010	0.1316	0.1604	0.1594	0.0004	0.0168
	Green	−3.93 × 10^−4^	0.2018	0.1486	0.1681	0.1717	−0.0233	0.0127
	Red	−4.36 × 10^−4^	0.2247	0.1822	0.1960	0.2057	−0.0223	0.0103
	NIR	−5.04 × 10^−4^	0.2862	0.2262	0.2510	0.2508	−0.0282	0.0128
